# Association of Pre-Treatment Nutritional Status with Change in CD4 Count after Antiretroviral Therapy at 6, 12, and 24 Months in Rwandan Women

**DOI:** 10.1371/journal.pone.0029625

**Published:** 2011-12-28

**Authors:** Elizabeth Kiefer, Donald R. Hoover, Qiuhu Shi, Jean-Claude Dusingize, Mardge Cohen, Eugene Mutimura, Kathryn Anastos

**Affiliations:** 1 Montefiore Medical Center, Bronx, New York, United States of America; 2 Albert Einstein College of Medicine, Bronx, New York, United States of America; 3 Institute for Health, Health Care Policy and Aging Research, Rutgers University, Piscataway, New Jersey, United States of America; 4 New York Medical College, Valhalla, New York, United States of America; 5 John Stroger (formerly Cook County) Hospital and Rush University, Chicago, Illinois, United States of America; 6 Women's Equity in Access to Care and Treatment, Kigali, Rwanda; University of Cape Town, South Africa

## Abstract

**Background:**

Body mass index (BMI) independently predicts mortality in studies of HIV infected patients initiating antiretroviral therapy (ART). We hypothesized that poorer nutritional status would be associated with smaller gains in CD4 count in Rwandan women initiating ART.

**Methods and Findings:**

The Rwandan Women's Interassociation Study and Assessment, enrolled 710 ART-naïve HIV-positive and 226 HIV-negative women in 2005 with follow-up every 6 months. The outcome assessed in this study was change in CD4 count at 6, 12, and 24 months after ART initiation. Nutritional status measures taken prior to ART initiation were BMI; height adjusted fat free mass (FFMI); height adjusted fat mass (FMI), and sum of skinfold measurements. 475 women initiated ART. Mean (within 6 months) pre-ART CD4 count was 216 cells/µL. Prior to ART initiation, the mean (±SD) BMI was 21.6 (±3.78) kg/m^2^ (18.3% malnourished with BMI<18.5); and among women for whom the following were measured, mean FFMI was 17.10 (±1.76) kg/m^2^; FMI 4.7 (±3.5) kg/m^2^ and sum of skinfold measurements 4.9 (±2.7) cm. FFMI was significantly associated with a smaller change in CD4 count at 6 months in univariate analysis (−6.7 cells/uL per kg/m^2^, p  = 0.03) only. In multivariate analysis after adjustment for covariates, no nutritional variable was associated with change in CD4 count at any follow up visit.

**Conclusion:**

In this cohort of African women initiating ART, no measure of malnutrition prior to ART was consistently associated with change in CD4 count at 6, 12, and 24 months of follow up, suggesting that poorer pre-treatment nutritional status does not prevent an excellent response to ART.

## Introduction

HIV-infection and poor nutritional status are prevalent in Africa. In Rwanda, malnutrition defined by the World Health Organization (WHO) definition of body mass index (BMI) < 18.5 kg/m^2^, [1] is substantially greater than in developed countries [2]; in our preliminary studies of Rwandan HIV-positive women in 2005 approximately 19% were malnourished [3].

Low body mass index (BMI) and serum albumin have been shown to independently predict mortality in HIV infected persons initiating ART in several United States and African studies [4], [5], [6], [7], [8], [9]; and also may be markers of advanced HIV disease [5], [10]. In an Ethiopian study, weight loss in the first month after antiretroviral treatment (ART) initiation was associated with shorter survival [6]. In the Tufts Nutrition for Healthy Living Cohort as little as 5% weight loss over 6 months or 3% from baseline, was associated with increased mortality in HIV infected persons, even after adjustment for ART use [8]. Malnutrition can lead to increased susceptibility to infection through suppression of immune defense mechanisms, and HIV infection can cause malnourishment through increased concomitant opportunistic infections, malabsorption, and anorexia. However, dietary supplementation with food, which could potentially increase weight and improve nutritional status, has failed to consistently show reductions in HIV mortality in both high- and low-resource settings [11], [12]. It is thus unclear whether nutritional measures such as BMI are simply markers of more progressive HIV disease and thus impending mortality, or are functioning independently as causal factors for illness or death; these questions have important programmatic and policy implications for food supplementation for HIV infected persons.

If CD4 cell response to ART were lower in malnourished compared to well-nourished individuals, this might imply a causal role of malnutrition in HIV mortality. However, few studies have investigated the association of malnutrition and CD4 response. Those that did used only BMI or body weight as a marker of malnutrition, which will not separately capture the different fat and non-fat (i.e. protein) aspects of malnutrition.. In the Singapore HIV Observational Cohort, there was no association of BMI measured 2 months before or after ART initiation with the magnitude of CD4 increase at 6 and 12 months after ART initiation, although lower BMI was significantly associated with reduced survival [13]. Among HIV infected patients initiating ART in Cote d'Ivoire there was no difference in the proportion of people who gained 50 or more CD4 cells/uL 6 months after ART initiation among BMI categories >25, 18.5–25, and <18.5 kg/m^2^ [14].

BMI may provide an incomplete picture of pre-ART nutritional status in HIV infected individuals. BMI is an easily measured and well-validated indicator of malnutrition in populations without HIV, but it may not capture the changes in body composition that occur specifically in HIV infection because it does not distinguish fat from protein. Starvation and cachexia are two pathologically different forms of malnutrition that may occur in HIV-infected populations; whereas starvation involves losses in body fat, cachexia results in mainly a loss of lean body mass such as skeletal muscle [14]. Further, in cachexia, changes in inflammation and the acute phase response leads to an elevation in resting energy expenditure and hypermetabolism, which in turn may affect response to ART [15]. Therefore, height-normalized measures of fat-free mass and fat mass may provide important information about specific fat and protein malnutrition within these different compartments in persons with HIV infection [16]. Skinfold measurements may also capture malnutrition by providing an estimate of subcutaneous fat. These measures have been validated as nutritional markers in non-African populations with HIV [17]. In this cohort we previously showed correlation between these measures (r^2^ = .48), and several small studies have shown high correlation (r^2^ = .61 to .87) between fat mass and fat free mass between calculated and anthropometric measures of body fat, although among Africans of unknown HIV serostatus [18], [19], [20].

We therefore examined the association of nutritional status prior to ART initiation, using height-normalized measures of body mass, fat-free mass, fat mass, and skin fold thickness, with the change in CD4 count at an average of 6, 12, and 24 months after ART initiation in a cohort of Rwandan women with a high prevalence of malnutrition at study entry.

## Methods

### Objectives

The objectives of this study were to examine the association of nutritional status prior to ART initiation, using height-normalized measures of body mass, fat-free mass, fat mass, and skin fold thickness, with the change in CD4 count at an average of 6, 12, and 24 months after ART initiation in a cohort of Rwandan women with a high prevalence of malnutrition at study entry. We hypothesized that poorer nutritional status would be associated with smaller gains in CD4 count in Rwandan women initiating ART.

### Participants

The Rwanda Women's Interassociation Study and Assessment (RWISA) is an observational prospective cohort of 710 ART-naïve (at enrollment) HIV-infected and 226 HIV-uninfected Rwandan women enrolled in 2005 [21]. Briefly, participants met the following inclusion criteria: age 25 years or older at study entry, willingness to give informed consent, presence in Rwanda during 1994. Participants were excluded if there was a prior history of receiving antiretroviral treatment other than single-dose nevirapine to prevent mother-to-child transmission of HIV. At study entry and at six-month interval visits, participants provided historical information including socio-demographics, medical history and symptoms, anthropometric measurements and blood specimens.

At each follow up visit, participants were asked whether they had initiated ART. Women reporting ART initiation provided written documentation of the exact date of ART initiation and medication regimen. From 2005 through 2007, following WHO and Rwandan guidelines, women in this cohort were eligible for ART if they had: WHO Stage IV disease, irrespective of CD4 cell count; WHO Stage III disease with CD4 <350 cells/µL; or CD4 <200/µL regardless of clinical stage. In 2008 Rwandan guidelines expanded the CD4 criterion to include ART initiation in all patients with CD4<350 cells/µl.

Included in this analysis are all HIV-positive RWISA participants who initiated ART after study entry, had a CD4 count obtained within one year prior to ART initiation (pre-ART CD4 count), nutritional measures at the pre-ART CD4 visit, and at least 6 months of follow-up with a CD4 measurement.

### Body composition and anthropometric measurements

Height and weight were measured while the participant was wearing light clothing and no shoes. Body impedance analysis (BIA) was performed twice using a standard tetrapolar electrode placement on the hand and foot with resistance and reactance recorded. Skin-fold measurements were obtained by study nurses who were trained on techniques for standardized anthropometric measurement, using correct anatomic location of skin folds.

### Laboratory Methods

CD4 counts were determined with a FACS counter (Becton and Dickinson, Immunocytometry Systems, San Jose, CA, USA).

### Outcome variables

The main outcome of interest was change in CD4 count from the pre-ART to the 6, 12, and 24 month follow-up visits. Pre-ART values were defined as those measured at the study visit which fell between 1 day and 12 months prior to the exact date of ART initiation. If there were multiple visits in this time window, we chose the date closest to the exact date of ART initiation. The 6-, 12- and 24-month follow up visits were defined as the study visits which fell between 3 and 9 months, 9 and 15 months, and 21 and 27 months respectively after the exact date of ART initiation. Because some participants did not complete their follow-up at exact 6 month intervals, it was possible to have more than one visit in the follow up range, in which case the visit closest to the 6, 12, or 24 month follow up date was used.

The change in CD4 was calculated as the absolute CD4 count at the follow up visit minus the pre-ART CD4 count. Since the pre-ART CD4 count visit was on average 91 days (3 months) prior to the exact date of ART initiation, the change in CD4 at 6 months reflects on average a change over 9 months (with on average 6 of these months on ART). The change in CD4 at 12 months reflects on average, a change over 15 months (with on average 12 months on ART), and the change in CD4 at 24 months reflects on average a change over 27 months (with on average 24 months on ART).

### Nutritional predictor variables

Four nutritional indicators were used, all from the pre-ART visit: body mass index (BMI); fat free mass index (FFMI); fat mass index (FMI); and the sum of skinfold measurement at the mid triceps, front thigh, and sub-scapular regions.

BMI, obtained from standing height and weight measurements, was calculated as weight divided by height-squared (kg/m^2^). We used WHO-established BMI cutoffs for nutritional status: malnourished (BMI < 18.5 kg/m^2^), normal (BMI 18.5–25 kg/m^2^), and overweight (BMI≥25 kg/m^2^) [1].

FFMI was obtained from BIA as described above. Resistance and reactance were entered into standard formulae to calculate fat free mass in kg; these formulae have been previously validated in a multi-ethnic HIV and non-HIV population in the United States, and have been used in several African-studies [17], [22], [23], [24], [25]. For <5% of the participants the calculated FFM exceeded weight and was thus set to equal the weight. We then standardized the calculated fat free mass by dividing it by height in meters, squared, to obtain the FFMI (kg/m^2^). The FMI was calculated as weight in kg minus fat free mass in kg, and was standardized for height by dividing by height-squared (kg/m^2^).

Skinfolds measurements were taken twice by the same person at each site of mid triceps, front thigh, and sub-scapular region, and the average of the two measurements was used for analysis. If the two measurements differed by ≥2.0 mm, a third measure was taken and the closest two were averaged. The sum of the skinfolds measurement was the sum of the triceps, thigh and sub-scapular measurements in centimeters.

### Covariates

We included the following covariates obtained at the pre-ART visit because of their potential to confound the relationship between malnutrition and change in CD4 count: age, income, education, CD4 count, and self-reported prior occurrence of a Stage 4 WHO AIDS defining illness (ADI). Smoking was not included in the analysis as less than 3% of the women smoked.

Age was included as age per 5 years. Income was categorized as <10,000 Rwandan francs (FRW) per month (in 2005, this was equivalent to < $17), 10,000 to 35,000 FRW per month, and greater than 35,000 FRW per month. Education was categorized as none, some primary school, completed primary school, or some secondary school or higher. Pre-ART CD4 count was included in the analysis per 100 cells/µL increment. We determined the presence of Stage 4 WHO illness from participant self-report at all visits prior to and including the pre-ART study visit.

### Ethics

Each participant provided written informed consent after viewing a video demonstrating study procedures, and discussing the study with research personnel. Study protocols were approved by the Rwanda National Ethics Committee and the Institutional Review Board of Montefiore Medical Center.

### Statistical Methods

The primary outcomes were changes in CD4 count (as a continuous variable), from the pre ART visit at 6, 12, and 24 months of follow up, as defined above. Univariate linear regression analysis was performed for the change in CD4 count at the 6-, 12-, and 24-months post ART. All nutritional measures were analyzed as continuous variables. Other covariates were analyzed as described above.

For the multivariate analysis, we created four multivariate linear regression models each containing one of the nutritional variables alone in the model (BMI, FFMI, FMI or skinfold sum) with the covariates listed above. This was performed 3 times using change from pre-ART visit to 6, 12, and 24 months post-ART as the outcome. We used backwards selection analysis with a p-value of p = 0.1 to stay. Variables that did not reach statistical significance were removed from the model until we obtained a final model. SAS software, version 9.1.3 (Cary, North Carolina) was used for the analysis.

## Results

Table 1 displays demographic and clinical characteristics of the 537 included women. The median preART CD4 count was 200 (interquartile range 142 to 279) cells/µL, The mean time from pre-ART measurement to the 6 month follow up visit was 244 days (8.0 months) with a median of 210 days (6.9 months); to the 12 month follow up visit, 425 days (14.0 months) with a median of 390 days (12.8 months); and to the 24 month follow up visit, 777 days (25.6 months) median of 771 days (25.3 months).

**Table 1:** Characteristics of 537 HIV-infected women participants prior to initiation of antiretroviral therapy.

Variable	Mean±SD or N(%)
**Demographic variables**	
Age, years (n = 537)	35.2±6.9
Income, RWF/year (n = 525)	
Income <10,000	192 (36.6%)
Income 10,000–35,000	266 (50.7%)
Income >35,000	67 (12.8%)
Education (n = 531)	
None	118 (22.2%)
Some primary school	200 (37.7%)
Completed primary school	156 (29.4%)
Some secondary or higher	57 (10.7%)
CD4 count, cells/µL (n = 537)	216±114
CD4 < 200	269 (50.1%)
CD4 200–350	212 (39.5%)
CD4 >350	56 (10.4%)
WHO Stage 4 illness prior to ART (n = 224)	224 (41.7%)
**Nutritional variables**	
BMI, kg/m^2^ (n = 526)	21.6±3.8
BMI < 18.5	96 (18.3%)
BMI 18.5–20	95 (18.1%)
BMI >20	335 (63.7%)
FFMI, kg/m^2^ (n = 463)	17.1±1.8
FMI, kg/m^2^ (n = 463)	4.7±3.5
Skinfold sum, cm (n = 489)	4.9±2.7

Abbreviations: RWF = Rwandan Francs; WHO =  World Health Organization; BMI = body mass index; FFMI = fat free mass index; FMI = fat mass index.

Prior to ART initiation, the mean (±Standard Deviation) BMI was 21.6 (±3.8) kg/m^2^, with 18.3% of the women classified as malnourished (BMI<18.5). The mean pre-ART FFMI and FMI were 17.1 (±1.8) kg/m^2^ and 4.7 (±3.5) kg/m^2^ respectively. The sum of these means (17.1 kg/m^2^ + 4.7 kg/m^2^) does not equal the mean BMI (21.6 kg/m^2^), as some people with BMI were missing BIA measures and thus did not have a calculated FMI and FFMI. The mean pre-ART sum of the three skinfolds measurements was 4.9 (±02.7) cm.

The mean changes in CD4 count from pre ART initiation at 6, 12, and 24 months post initiation were 71 (±107), 89 (±109) and 153 (±135) cells/µL, respectively. In univariate linear models (Table 2), the only significant association (at p < 0.05) between any nutritional measure and pre-post ART change in CD4 count occurred at 6 months of follow up. A higher FFMI was associated with a smaller increase in CD4 count at 6 months (−7 cells/µL per kg/m^2^, p  = 0.03). For example, those with a FFMI of 17 kg/m^2^ gained on average 7 fewer CD4 cells/µL from the pre-ART visit to the 6 month post-ART visit compared to those with a FFMI of 16 kg/m^2^. BMI, FMI and skinfold measurements were not associated with change in CD4 count at 6 months. The changes in CD4 count at 12 and 24 months were not significantly associated with any of the pre-ART nutritional measurements in univariate analysis.

**Table 2:** Univariate variables and Change in CD4 count at 6, 12, and 24 months from pre-ART visit.

Variable	Change in CD4 count, cells/µL from pre-ART visit								
	6 month change			12 month change			24 month change		
	n	estimate	p	n	estimate	p	n	estimate	p
**Nutritional variables**									
BMI, per *kg/m^2^*	466	0.24	0.86	391	−1.5	0.35	294	−0.10	0.96
FFMI, per *kg/m^2^*	406	−6.7	**0.03**	330	−.44	0.89	253	−6.9	0.15
FMI, per *kg/m^2^*	406	1.8	0.28	330	−1.2	0.51	253	2.2	0.46
Skinfold sum, per c*m*	432	−0.40	0.83	353	−2.0	0.29	271	−3.3	0.19
**Demographic variables**									
Age (per 5 years)	475	−1.4	0.69	397	−3.1	0.43	296	−1.5	0.80
Income, RWF/year	475			397			296		
<10,000 baseline		Baseline			Baseline			Baseline	
10,000–35,000		−10	0.34		−26	**0.03**		−41	**0.02**
>35,000		−25	0.12		−26	0.13		−16	0.51
Education	475			397			296		
No education baseline		Baseline			Baseline			Baseline	
Some primary		−14	0.30		−7	0.61		−6.2	0.77
Completed primary		−13	0.36		−19	0.22		−9.5	0.66
Some secondary or higher		−59	**0.001**		−31	0.13		−15	0.61
Pre-ART CD4 count, per 100 cells/ul	475	−21	**<0.0001**	397	−13	**<0.0001**	296	−27	**<0.0001**
WHO Stage IV illness prior to ART	475	−1.5	0.88	397	22	0.05	296	−9.6	0.55

Abbreviations: ART = antiretroviral therapy, BMI = body mass index, FFMI = fat free mass index, FMI = fat mass index, RWF =  Rwandan Francs, WHO = World Health Organization.

A higher pre-ART CD4 count was strongly and significantly associated in univariate analysis with smaller CD4 increases at all timepoints (p<0.0001 for all) perhaps in part reflecting regression to the mean [26]. Of the demographic variables, at 6 months of follow up the only statistical association was with educational attainment: those having secondary school or higher compared to no education had a smaller increase in CD4 count (p = 0.001). At 12 and 24 months of follow up, an income of 10,000 to 35,000 FRW was associated with a smaller increase in CD4 count (p = 0.03 and p = 0.02, respectively).

In the four backwards selection models (BMI, FFMI, FMI, and sum of the skinfolds), no nutritional variable met criteria to stay in the adjusted models and thus none was independently associated with a change in CD4 cell count from pre-ART to 6, 12, or 24 months post-ART after adjustment for covariates (data not shown). In particular FFMI, which was associated with change in CD4 count at 6 months in univariate analysis, was no longer independently associated with this outcome in multivariate analysis. In all multivariate models preART CD4 remained independently inversely associated with change in CD4 at all time points (range from −13 and −36 cells/µl per 100 cells/µl increment; p = .01 to p<0.0001).

## Discussion

We did not find any association between BMI, FFMI, FMI or skinfolds measurements and the change in CD4 count from the pre-ART visit to 6, 12, or 24 months after ART initiation. In our study, we examined not only BMI, but a panel of anthropometric measures through the use of FFMI, FMI, and skinfolds to better capture pre-ART nutritional status in terms of loss of fat or protein within these body compartments. We found that none of these measures predicted change in CD4 count at any time point after initiation of ART, especially after adjustment for other known predictors of CD4 response such as pre-ART CD4 count, HIV viral load, or history of WHO Stage 4 AIDS defining illness. Thus, having a low BMI, low fat mass, low fat free (protein) mass or thin skinfolds did not preclude a robust response to ART as measured by the change in CD4 count. These multiple measures of either fat or protein loss, such as experienced in starvation or cachexia, were not associated with the ability to gain CD4 cells at several post-ART timepoints. In fact, the only statistically significant association we observed in univariate analysis (FFMI with CD4 change at 6 months) was in the opposite direction, suggesting a less robust response with higher FFMI. These anthropometric findings are similar to the findings from by Paton, Koethe and Toure, who examined only pre-treatment BMI and various responses to CD4 count and found no associations. To our knowledge, this is the first study to examine several anthropometric pre-ART markers of nutritional status and their associations with changes in CD4 count, particularly in African subjects.

It is possible that our measures may not have accurately captured nutritional status prior to treatment. However, our recent study of this cohort has shown that serum albumin levels, traditionally thought of as a marker of nutritional status, also did not correlate well with FMI, FFMI, BMI, or skinfolds [27]. Serum albumin was lowest in the lowest CD4 count strata, and thus was more likely a marker of disease severity than of nutritional status.

### Limitations

Some limitations to this study should be noted. Although the exact dates of ART initiation were known, the time from the pre-ART nutritional measurements to follow up visit varied. However, our results show that the time from the pre-ART measurements to follow up visits was generally within 1–2 months of the goal follow up at 6, 12, and 24 months. Another limitation is that the resistance and reactance measurements, obtained from BIA measurements, were used to calculate fat mass and fat free mass in equations derived from a white and African-American population which may not completely reflect the relationships in Rwanda [17], although these equations have been used in several published studies in Africa [22], [23], [24], [25]. We also note inter-operator variability when obtaining skinfold measurements, although we took care with multiple measurements for accuracy by our trained nurses. We were unable to define fully from participant self-report all WHO Stage 4 AIDs defining illnesses prior to initation of ART, as some of these illnesses were not specifically inquired about; thus we may not have accurately captured all confounding illness. Finally, caution must be made in extrapolating these data to populations with different levels of malnutrition.

### Conclusions

In summary, we found that pre-ART nutritional status, as measured by BMI, FFMI, FMI and skinfolds did not predict the pre to post ART initiations changes in CD4 count at 6, 12, and 24 months after ART initiation in a cohort of HIV infected Rwandan women. Thus having poor values on these anthropometric measures may not preclude an excellent response to ART as measured by CD4 count. Further studies are warranted, using additional measures of nutritional status and with longer follow-up time, to assess further the impact of malnutrition on surrogate markers and clinical outcomes including mortality in Africans initiating ART.
